# Exosome-transported circRNA_0001236 enhances chondrogenesis and suppress cartilage degradation via the miR-3677-3p/Sox9 axis

**DOI:** 10.1186/s13287-021-02431-5

**Published:** 2021-07-13

**Authors:** Guping Mao, Yiyang Xu, Dianbo Long, Hong Sun, Hongyi Li, Ruobin Xin, Ziji Zhang, Zhiwen Li, Zhi Yang, Yan Kang

**Affiliations:** 1 Department of Joint Surgery, First Affiliated Hospital of Sun Yat-sen University, #58 Zhongshan 2nd Road, Guangzhou, 510080 Guangdong China; 2 Guangdong Provincial Key Laboratory of Orthopedics and Traumatology, Guangzhou, China; 3 Department of Orthopedics, Fujian Provincial Hospital/Shengli Clinical Medical College, Fujian Medical University, Fuzhou, 350003 Fujian China; 4 Department of Orthopaedics, Affiliated Hospital of Guizhou Medical University, Guiyang, 550004 Guizhou China

**Keywords:** Osteoarthritis, Exosome, circRNA, Chondrogenesis, Chondrocytes

## Abstract

**Objectives:**

Aberrations in exosomal circular RNA (circRNA) expression have been identified in various human diseases. In this study, we investigated whether exosomal circRNAs could act as competing endogenous RNAs (ceRNAs) to regulate the pathological process of osteoarthritis (OA). This study aimed to elucidate the specific MSC-derived exosomal circRNAs responsible for MSC-mediated chondrogenic differentiation using human bone marrow-derived MSCs (hMSCs) and a destabilization of the medial meniscus (DMM) mouse model of OA.

**Methods:**

Exosomal circRNA deep sequencing was performed to evaluate the expression of circRNAs in human bone marrow-derived MSCs (hMSCs) induced to undergo chondrogenesis from day 0 to day 21. The regulatory and functional roles of exosomal circRNA_0001236 were examined on day 21 after inducing chondrogenesis in hMSCs and were validated in vitro and in vivo. The downstream target of circRNA_0001236 was also explored in vitro and in vivo using bioinformatics analyses. A luciferase reporter assay was used to evaluate the interaction between circRNA_0001236 and miR-3677-3p as well as the target gene sex-determining region Y-box 9 (*Sox9*). The function and mechanism of exosomal circRNA_0001236 in OA were explored in the DMM mouse model.

**Results:**

Upregulation of exosomal circRNA_0001236 enhanced the expression of Col2a1 and Sox9 but inhibited that of MMP13 in hMSCs induced to undergo chondrogenesis. Moreover, circRNA_0001236 acted as an miR-3677-3p sponge and functioned in human chondrocytes via targeting miR-3677-3p and *Sox9*. Intra-articular injection of exosomal circRNA_0001236 attenuated OA in the DMM mouse model.

**Conclusions:**

Our results reveal an important role for a novel exosomal circRNA_0001236 in chondrogenic differentiation. Overexpression of exosomal circRNA_0001236 promoted cartilage-specific gene and protein expression through the miR-3677-3p/Sox9 axis. Thus, circRNA_0001236-overexpressing exosomes may alleviate cartilage degradation, suppressing OA progression and enhancing cartilage repair. Our findings provide a potentially effective therapeutic strategy for treating OA.

**Supplementary Information:**

The online version contains supplementary material available at 10.1186/s13287-021-02431-5.

## Background

Osteoarthritis (OA) is the most prevalent chronic joint disease representing a substantial and increasing health burden with notable implications for the affected individuals and greater socioeconomic costs [1, 2]. OA is a whole joint disease involving structural alterations in the hyaline articular cartilage, subchondral bone, ligaments, capsule, synovium, and periarticular muscles [3]. OA’s pathogenesis involves mechanical, inflammatory, and metabolic factors that ultimately lead to structural destruction and failure of the synovial joint [4]. It involves an active dynamic alteration arising from an imbalance between the repair and destruction of the joints. The extracellular matrix (ECM) consists of type II collagen (collagen II) to maintain joint tissue homeostasis. Previous studies have indicated that overexpression of SOX9 can promote cartilage repair and can be used as a potential therapeutic agent at the early stages of human OA [5]. Moreover, Sox9 is essential for the expression of col2a1, which plays an important role during chondrogenesis [5]. ECM degeneration during OA is regulated by growth factors, inflammatory factors, and non-coding RNAs (ncRNAs); therefore, it is important to elucidate ECM degeneration’s mechanism during OA [6, 7].

Exosomes are microvesicles with a 40-150 nm diameter that can carry various proteins, lipids, and nucleic materials, such as DNA, RNA, messenger RNA (mRNA), and ncRNA [8, 9]. Several studies have shown that exosomes originate from multivesicular endosomes by inverse budding to form multivesicular bodies and are released into the extracellular space when a multivesicular body fuses with the plasma membrane [10]. Thus, exosomes deliver cargo from recipient cells to regulate pathophysiological processes [9, 11], including immune responses [12–14], inflammation, tumor growth [15, 16], and infection [17, 18], and are produced and secreted by several cell types [19, 20]. Mesenchymal stem cell (MSC)-derived exosomes can deliver nucleic acids, proteins, and lipids to provide a favorable microenvironment that enhances cartilage repair and delays OA progression [21, 22]. Circular RNAs (circRNAs) are a family of covalently closed ncRNA molecules produced by the back splicing of exons in precursor mRNAs of eukaryotes [23]. Current studies have shown that circRNAs are closely involved in OA occurrence and repair [24, 25]. Although a growing number of studies have demonstrated stable circRNAs in exosomes [26, 27], the role that MSC-derived exosomal circRNAs have in regulating chondrogenesis and cartilage degradation remains unknown.

This study aimed to elucidate the specific MSC-derived exosomal circRNAs responsible for chondrogenic differentiation through circRNA microarray analyses of hMSCs. We identified circZC3H7B (hsa_circ_0001236) as an important exosomal circRNA during chondrogenesis in MSCs. Given the role of MSC-derived exosomal circRNAs in regulating cartilage repair and homeostasis, we hypothesized that exosomal circZC3H7B might contribute to both chondrogenic differentiation and OA pathogenesis by regulated *S*ox9 expression. We further aimed to determine the mechanism underlying MSC-derived exosomal circZC3H7B’s action on cartilage differentiation and maintenance.

## Methods

### Samples, MSCs culture, and chondrogenesis

Bone marrow samples were procured from the First Affiliated Hospital of Sun Yat-sen University after obtaining written informed consent from all donors. Bone marrow samples were obtained by iliac crest aspiration of six healthy human donors (mean age, 36 years; range, 32–38 years; male, *n* = 3). hMSCs were isolated as described previously [28, 29]. Cells were cultured in a basal medium [alpha-modified Eagle’s medium (α-MEM); Gibco Life Technology, Grand Island, NY, USA] supplemented with 10% fetal bovine serum (FBS). When cultures neared 80% confluence, cells were detached by treatment with 0.05% trypsin/ethylenediaminetetraacetic acid (EDTA) and passaged in culture. All hMSCs were used at passage 3 to induce hMSC chondrogenesis by micromass culture, as previously described [28]. Briefly, hMSCs were resuspended at 2 × 10^7^ cells/mL in the incomplete chondrogenic medium [97 mL human mesenchymal stem cell chondrogenic differentiation basal medium, 10 μL dexamethasone, 300 μL ascorbate, 1 mL of ITS (insulin, transferrin, selenium) supplement, 100 μL sodium pyruvate, and 100 μL proline; Cyagen Biosciences, Guangzhou, China]. Micromass (droplets) of resuspended cells (12.5 μL) were carefully transferred to individual wells of a 24-well plate and incubated at 37 °C for 90 min to stimulate the adherence of cells to the plate. Micromass was cultured in 500 μL complete chondrogenic induction medium, prepared by the addition of 10 μL transforming growth factor (TGF)-β3 to 1 mL incomplete chondrogenic medium (Cyagen Biosciences). Samples and supernatant were collected for experiments at selected time points.

### Isolation and identification of exosomes

Exosome isolation was carried out by ultracentrifugation as previously described [28]. In brief, MSC culture supernatants were subjected to successive centrifugations at 3000×*g* (30 min) and 10,000×*g* (30 min). Exosomes were then pelleted at 64,000×*g* for 110 min using an SW28 rotor (Beckman Coulter, USA). Exosome pellets were resuspended in 0.32 M sucrose and centrifuged at 100,000×*g* for 90 min (SW60Ti rotor, Beckman Coulter). The exosome pellet was resuspended in phosphate-buffered saline (PBS). Nanosight 2000 (nanoparticle tracking analysis (NTA), Malvern, UK) analysis and transmission electron microscopy (TEM) were used to identify exosomes. RNA and proteins were extracted from exosomes using a Total Exosome RNA & Protein Isolation Kit (Invitrogen, Carlsbad, CA, USA) for further analysis.

### Primary chondrocytes collection, isolation, and culture

Degraded joint cartilage samples were obtained from patients with OA [*n* = 6; mean age, 58.22 years; male, *n* = 3, female, *n* = 3] during total knee replacement operations. Normal cartilage samples were taken from patients with no history of OA or rheumatoid arthritis. The patients underwent knee joint amputation because of traffic trauma (*n* = 6; mean age, 53.38 years; male, *n* = 3, female, *n* = 3). The cartilages were dissected from the subchondral bone and then digested by 4 mg/mL protease and 0.25 mg/mL collagenase P as described previously [30]. Cells were cultured in DMEM/F-12 (Gibco Life Technology) containing 5% FBS (Gibco Life Technology), 1% penicillin, and streptomycin (Gibco Life Technology). The chondrocytes were used for subsequent experiments within 3–7 days without passaging to avoid dedifferentiation.

### RNA extraction, reverse transcription, and quantitative real-time polymerase chain reaction (qRT-PCR)

RNA extraction and reverse transcription were performed as described previously [28]. Briefly, TRIzol Reagent (Thermo Fisher Scientific) was used to isolate cellular RNA, and the miRNeasy mini kit (QIAGEN, Hilden, Germany) was used to extract exosomal RNA. Following total RNA extraction, the first-strand cDNA was reverse transcribed using a PrimeScript RT reagent kit with genomic DNA (gDNA) Eraser (Takara, Nojihigashi, Japan), and miRNA inverse transcription was carried out using a Mir-X miRNA qRT-PCR SYBR kit (Clontech, Nojihigashi, Japan). qRT-PCR was performed on a Bio-Rad CFX96 using the PrimeScript RT reagent kit and SYBR Premix Ex Taq (TaKaRa) with the reaction conditions set as per the manufacturer’s instructions. Transcript levels were normalized to that of the housekeeping gene glyceraldehyde 3-phosphate dehydrogenase (GAPDH; for mRNA) or the small U6 RNA (for miRNA). Gene expression was calculated using the 2^−ΔΔCt^ method, and each experiment was performed in triplicate. The specific primers used for these analyses are listed in Supplementary Table 2.

### Competing endogenous RNAs (ceRNAs) analysis

The highest upregulated circRNA was selected based on exosome real-time PCR, and ceRNA analysis was carried out. A circRNA–miRNA–mRNA network was constructed to predict the possible interactions between the selected circRNA and sponged miRNAs using the Arraystar software, which was developed based on TargetScan and miRanda.

### Luciferase constructs and reporter assay

OBio Technology (Shanghai, China) synthesized circ_0001236, mutant circ_0001236, Sox9 wild-type 3′UTR, and Sox9 mutant 3′UTR cloned them into pMIR-REPORT vector between MluI and HindIII sites. Before transfection, HEK293T cells, at a density of 5 × 10^3^ cells/well, were cultured in 96-well plates for 24 h. Then, the cells were co-transfected with either pMIR-REPORT-circ_0001236, pMIR-REPORT-circ_0001236 mutant, pMIR-REPORT-Sox9-3′UTR, or pMIR-REPORT-Sox9-3′UTR mutant plasmid, inner control pRL-CMV Renilla luciferase plasmid (Promega, WI, USA), and miR-3677-3p (RiboBio, Guangzhou, China) at a final concentration of 100 nM. After incubation for 48 h, the cells were collected and processed according to the manufacturer’s protocol with the Dual-Luciferase Reporter Assay System (Promega). The outcomes were quantified in each well as the proportion of firefly luciferase/Renilla luciferase activity. A histogram was constructed to visualize the experimental results.

### Plasmid construction and stable transfection

To overexpress hsa-circ_0001236, the complete sequence of hsa-circ_0001236 was cloned into pcDNA3.1(+)-S-circRNA vector, containing a sequence with a circularization processing signal. Non-hsa-circ_0001236 vector was used as a control (NC; OBiO Technology). MSCs were transfected with miR-3677-3p mimic or inhibitor (RiboBio, Guangzhou, China) at a concentration of 50 nM; they were also transfected with pcDNA3.1(+)-S-hsa_circ_0001236 or NC. Lipofectamine®2000 (Gibco Life Technologies) was used to transfect cells according to the manufacturer’s instructions. Cells were harvested after 48 h for qRT-PCR or after 72 h for western blot analysis.

### RNA fluorescence in situ hybridization (FISH)

RiboBio (Guangzhou, China) engineered and synthesized specific probes for the circ_0001236 sequence used for FISH. The hybridization was carried out using the FISH kit (RiboBio) as per the manufacturer’s instructions using a Cy3-labeled circ_0001236 probe (5′-GCAGGGCTCTGGCTTTGCACAGGTAGTA-3′) and a digoxigenin (DIG)-labeled locked nucleic miR-3677-3p probe (5′-GGCCGTGGCCAGAGCCCACGAG-3′). Briefly, hybridization was carried out overnight at 37 °C in a humid chamber after washing, fixing, and permeating the cells. The nuclei were stained for 5 min with 4,6-diamidino-2-phenylindole (DAPI). Then, the cells were washed with cold PBS and assembled using antifade buffer (Beyotime, Shanghai, China). Images were captured and evaluated using a Zeiss LSM 880 NLO confocal microscope (Leica Microsystems, Wetzlar, Germany).

### Western blot analysis

Western blot analysis was carried out as described previously [30]. The membranes were incubated with primary antibodies against Sox9, COL2A1, MMP13 (1:1000, Abcam, Cambridge, UK), and GAPDH (1:3000, Cell Signaling Technology). Then, the blots were incubated with the corresponding secondary antibodies conjugated with HRP (1:3000, Cell Signaling Technology) at room temperature for 1 h. The protein bands were detected using ChemiDoc Touch (BIO-RAD, USA) and analyzed using Image LabTM (BIO-RAD, USA). The intensities of the bands were compared using the ImageJ software (https://imagej.en.softonic.com/).

### Immunohistochemical analysis

Immunohistochemical analysis was performed as described previously [28]. Briefly, the cartilage tissue sections were blocked in PBS containing 0.025% Tween 20 and 10% FBS, followed by incubation with rabbit anti-human Col2a1/MMP13 antibody (1:200 dilution; Abcam) and Sox9 (1:500, Millipore) overnight at 4 °C. The next day, secondary antibodies were added, and the samples were observed under a BX53 microscope (Olympus, Tokyo, Japan).

### Destabilization of the medial meniscus (DMM) mouse model of OA

All procedures were approved by the First Affiliated Hospital of Sun Yat-sen University ([2013]A-110) Animal Research Committee. We procured 25 8-week-old male wild-type (WT) C57BL/6J mice from GemPharmatech, Co. (Jiangsu, China) and housed them in pathogen-free conditions for experimentation at 12 weeks of age. The mice were fed a normal diet and had access to water. At 12 weeks, the mice were operated on to induce OA as described previously [31], after being anesthetized by isoflurane inhalation (2–3% isoflurane for anesthesia induction and 1.5–2% isoflurane for anesthesia maintenance). The mice were subjected to DMM surgery of the right knees to induce OA, with the left knee sham-operated on as control. Starting at 14 weeks, all mice were randomly divided into five groups (*n* = 5/group) based on treatment received to the right knee: sham, DMM, MSC-Exos (exosomes secreted by MSCs), MSC-circ_0001236-Exos (exosomes secreted by circRNA_0001236-overexpressing MSCs), and MSC-circ_0001236 + miR-3677-3p mimc-Exos. Mice from the DMM, MSC-Exos, MSC-circ_0001236-Exos, and MSC-circ_0001236 + miR-3677-3p mimic-Exos groups were administered multiple intra-articular injections once a week, for 6 weeks, of 10 μL saline, MSC-Exos(500 μg/ml), MSC-circ_0001236-Exos(500 μg/ml), and MSC-circ_0001236 + miR-3677-3p mimic-Exos(500 μg/ml) with a microliter syringe (Hamilton Company, 1702) and 5-mm 30-gauge needles (Hamilton Company, 7803-05). After 4 weeks (about 23 weeks), the mice were sacrificed, and the knee joint tissue was harvested for further analysis by immunohistochemistry (IHC). The inability to rise or ambulate was considered the humane endpoint in this in vivo study; if a mouse suffered severe OA and was unable to access food or water, the mouse was euthanized.

### Statistical analysis

All experiments were performed with at least three biological replicates. Data are expressed as the mean ± SD. The independent t-test and Mann–Whitney U-test were used to identify the differences between the cohorts in this study depending on whether the data were normally distributed or not. One-way analysis of variance (ANOVA) and Kruskal–Wallis tests were carried out for multiple group comparisons. Data analyses were performed using SPSS version 20 (IBM Corporation, Armonk, NY, USA). Statistical significance was set at *P* < 0.05. The circRNA microarray statistical analysis of the undifferentiated MSC-Exos and chondro-differentiated MSC-Exos groups was estimated by t-test. circRNAs having fold changes > 2 and *P* < 0.05 are selected as the significantly differentially expressed.

## Results

### Identification of MSC-Exos

**Fig. 1:**
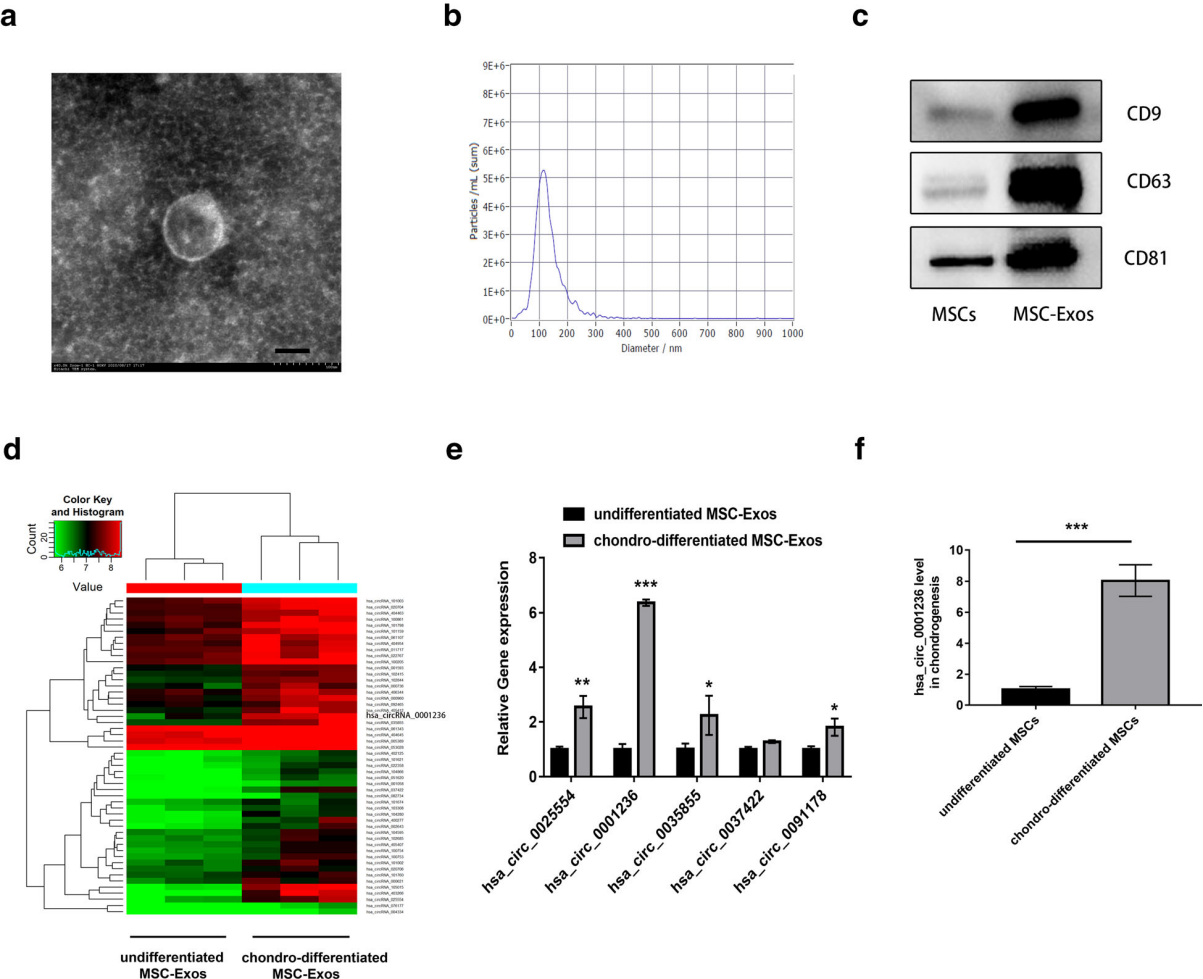
circRNA expression profile in the exosomes during the chondrogenesis of hMSCs. **a** Transmission electron microscopy showed the cup-shaped or round morphology of exosomes isolated from MSCs induced to undergo chondrogenesis. **b** The particle size and number of the exosomes were determined by nanoparticle tracking analysis (NTA). **c** Exosomal marker (CD9, CD63, CD81) protein expression of exosomes isolated from MSCs induced to undergo chondrogenesis. **d** Heat map showing significantly expressed circRNAs with ≥ 2-fold upregulation in MSCs that were induced to undergo chondrogenesis by TGF-β3 treatment for 0 and 21 days. Red and green colors indicate upregulated and downregulated circRNAs, respectively, with high fold change. **e**, **f** The top five most significantly differentially expressed exosomal circRNAs were verified. GAPDH was used as an internal control for circRNA expression. The data represent the mean ± SD of three independent experiments on samples from three different donors. *P < 0.05, **P < 0.01, ***P < 0.001. Scale bar, 100 nm

MSCs were aspirated from the iliac crest, isolated, and identified at passage 5 (P5) for subsequent experiments. TEM revealed that the MSC-Exos exhibited a cup-shaped or round morphology with a diameter of 50–150 nm (Fig. 1A). Moreover, Nanosight analysis showed that most MSC-Exos were approximately 50–150 nm in size (Fig. 1B). Western blot analyses indicated that the MSC-Exos expressed exosomal markers such as CD9, CD63, and CD81 (Fig. 1C).

### circRNA expression patterns in exosomes before and after chondrogenic differentiation of MSCs

The expression profiles of circRNAs in exosomes before and after chondrogenic differentiation were detected using a circRNA microarray. The Significance Analysis of Microarrays (SAM) statistical software was used to identify differentially expressed circRNAs between undifferentiated MSC-Exos and chondro-differentiated MSC-Exos. The differentially expressed circRNAs from all three paired samples are shown in Fig. 1D and Supplementary Table 1. Among the circRNAs that were consistently differentially expressed in all three paired samples, the top 12 circRNAs were upregulated (fold change ≥ 2) in chondro-differentiated MSC-Exos compared to undifferentiated MSC-Exos in Table 1. Among the top 5 most significantly differentially expressed exosomal circRNAs (Fig. 1E, F), exosomal circ_0001236 was upregulated 5.29-fold in exosomes after chondrogenic differentiation of MSCs. These data indicate that exosomal circRNAs play an important role in cartilage differentiation.

**Table 1:** Top 10 differentially expressed exosomal circRNAs (Chrond-exos vs normal exos)

circRNAs	Gene symbol	Fold change	*P*-value
hsa_circRNA_0025554	AEBP2	5.9168268	0.002053639
hsa_circRNA_0001236	ZC3H7B	5.2878911	0.000687383
hsa_circRNA_0035855	PIF1	3.2867378	0.009025781
hsa_circRNA_0037422	TSC2	2.8655818	0.024157636
hsa_circRNA_0091178	BRWD3	2.6619831	0.013174304
hsa_circRNA_0012265	GPBP1L1	2.6486815	0.006948549
hsa_circRNA_0039053	ITGAL	2.2715157	0.033519798
hsa_circRNA_0002643	NR4A3	2.2417031	0.000889744
hsa_circRNA_0082734	NDUFB2	2.225921	0.009542011
hsa_circRNA_0076177	SRPK1	2.1872971	0.025725088

### ceRNA network construction based on circRNA screening and bioinformatics prediction and Gene Ontology (GO) terms and KEGG pathway analysis identified key pathways regulated by exosomal circRNAs

**Fig. 2:**
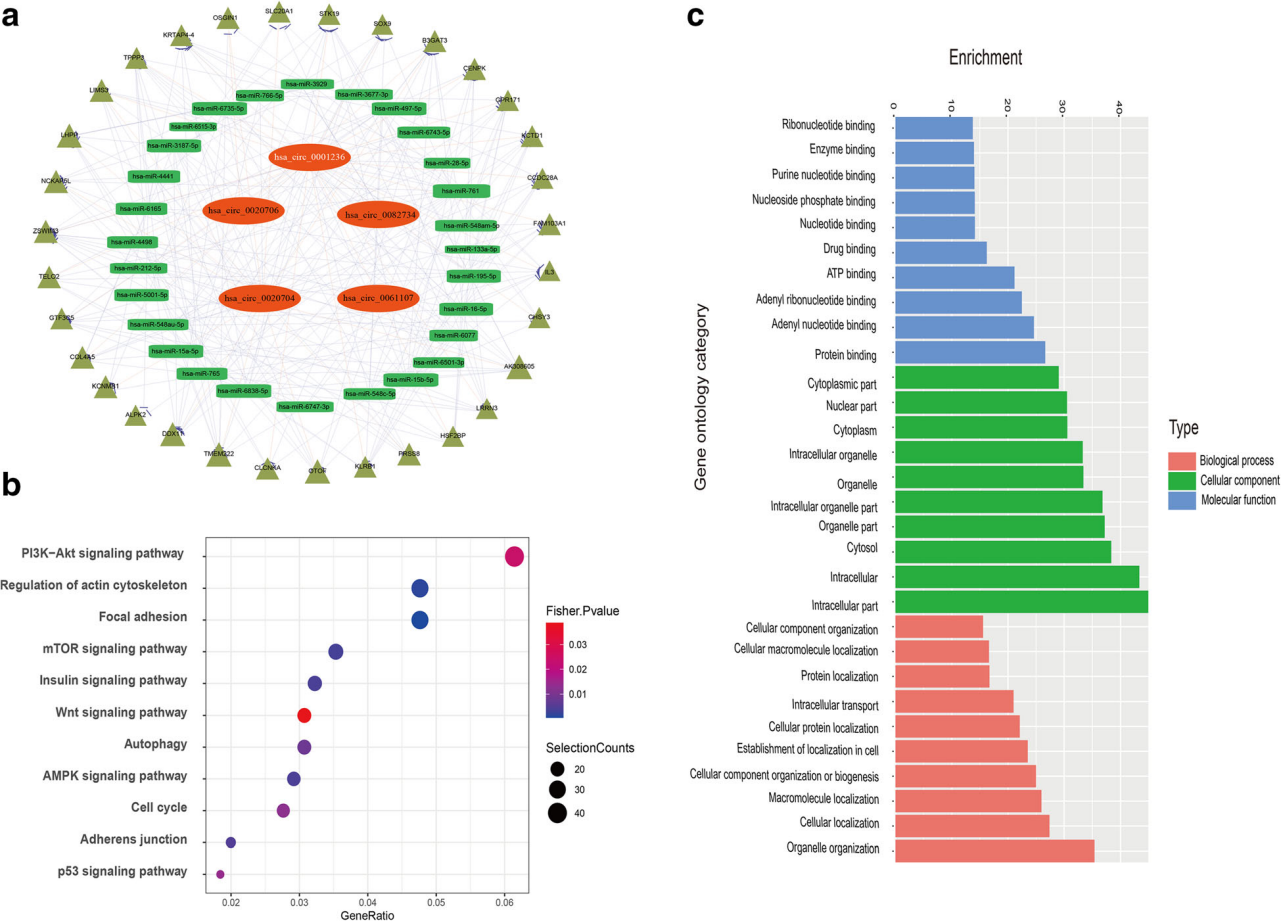
ceRNA network, GO terms, and KEGG pathway analysis of the screened exosomal circRNAs. **a** The cirRNA–miRNA–mRNA networks based on the selected five circRNAs, 28 miRNAs, and 31 mRNAs. **b** KEGG enrichment scatter plot for the target genes of the exosomal circRNAs. The exosomal circRNA target genes were grouped into gene pathways using KEGG enrichment analysis. The size of the circle is proportional to the number of enriched genes. KEGG, Kyoto Encyclopedia of Genes and Genomes. **c** GO enrichment summary of exosomal circRNA target genes. The GO enrichment analysis grouped the exosomal circRNA target genes into functional groups. The red, green, and blue columns represent the biological processes, cellular component, and molecular function, respectively. GO, Gene Ontology

The ceRNA regulatory mechanism between mRNA and ncRNAs, including miRNAs and circRNAs, is very important. We constructed the circRNA–miRNA–mRNA networks based on the selected 5 circRNAs, 28 miRNAs, and 31 mRNAs (Fig. 2A). These ceRNA regulatory relationships may be more important than previously thought in the process of chondrogenesis, considering the complexity of the ncRNAs and mRNAs. The GO enrichment analysis results revealed organelle organization in biological process, intracellular part in a cellular component, and protein binding in molecular function as significantly enriched terms for the target genes of differentially expressed exosomal circRNAs (Fig. 2B). In the KEGG pathway enrichment analysis, most exosomal circRNAs target genes were enriched in the PI3K–AKT signaling pathway, regulation of actin cytoskeleton, and focal adhesion (Fig. 2C).

### Characterization and expression analysis of exosomal circ_0001236

**Fig. 3:**
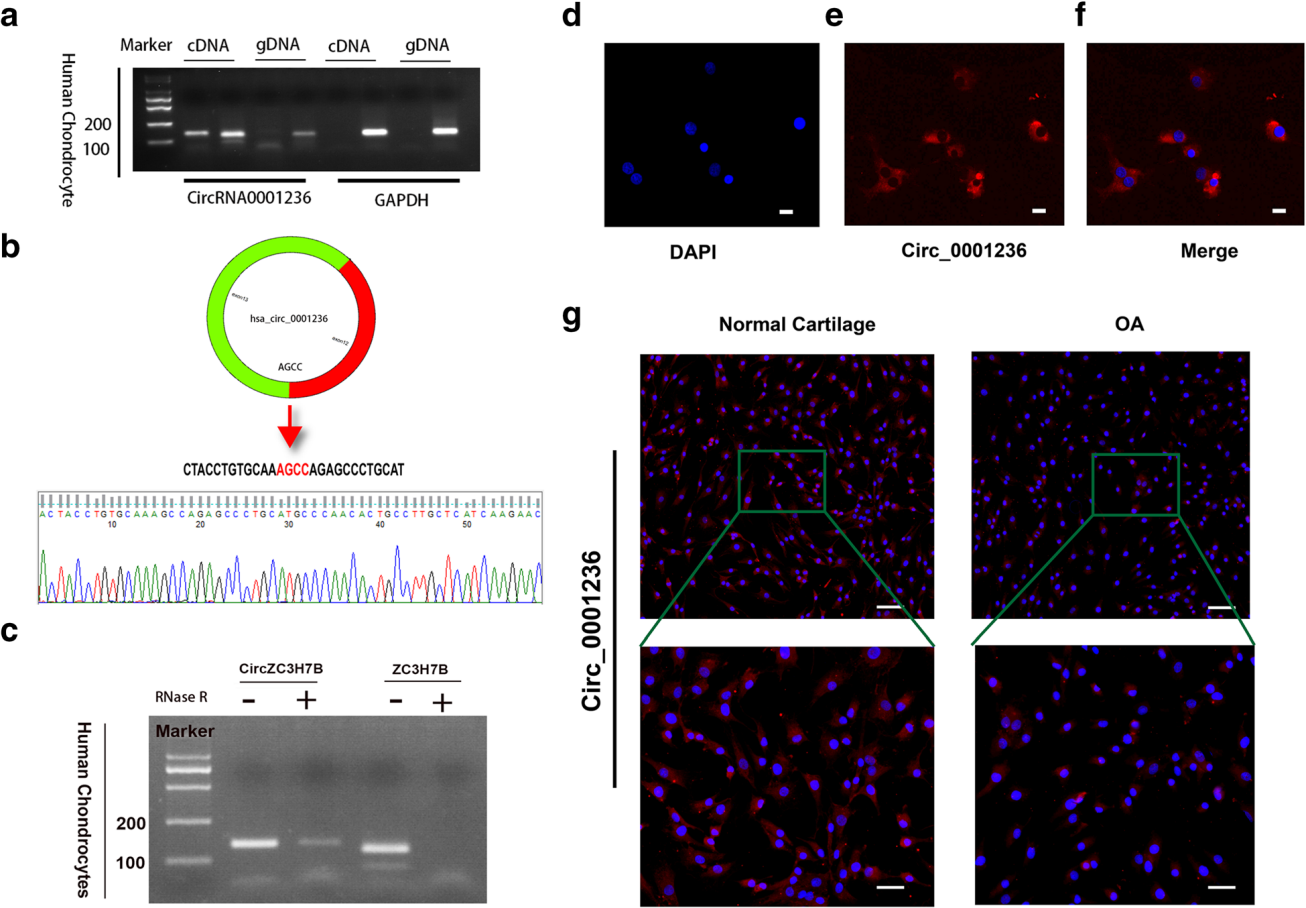
Exosomal circ_0001236 validation and expression in OA and healthy cartilage chondrocytes. **a** The presence of exosomal circ_0001236 was validated in primary human chondrocytes by RT-PCR. Divergent primers amplified circ_0001236 from cDNA, but not from genomic DNA. GAPDH was used as a negative control. **b** Schematic illustration showing the circularization of ZC3H78 exons 12 and 13 to form circZC3H78. The presence of circ_0001236 was validated by RT-PCR, followed by Sanger sequencing. **c** Northern blot analysis for the detection of circZC3H78 and ZC3H78 expression in chondrocytes treated with or without RNase R. **d**–**f** RNA FISH revealed the predominant localization of circ_0001236 in the cytoplasm. circ_0001236 probes were labeled with Cy-3. The nuclei were stained with DAPI (scale bar, 20 μm). **g** circ_0001236 expression was higher in healthy cartilage than in OA cartilage tissues (scale bar, 50 μm). The experiment was repeated independently three times, and representative results are shown

Our previous analysis focused on exosomal circ_0001236, which upregulated 5.29-fold during chondrogenic differentiation of MSC exosomes. We designed specific divergent primers for circ_0001236 (Table S2) and selected the circRNA as the template to perform PCR. PCR analysis yielded no products when using genomic DNA (gDNA) as the template (Fig. 3A). We found that exosomal circ_0001236 is spliced from the ZC3H7B gene (exons 12 and 13) on chr22:41738532|41739580(+), and the final length of circ_0001236 is 262 nt (Fig. 3B). Sanger sequencing of the PCR products amplified by divergent primers further confirmed the back-splice junction of circ_0001236 (Fig. 3B). These results showed that RT-PCR could specifically amplify the circRNA and confirmed the existence of circ_0001236 in chondrocytes. In the second step, we confirmed that circ_0001236 was resistant to RNase R, whereas ZC3H7B mRNA level was significantly decreased after RNase R treatment (Fig. 3C) as determined by northern blot analysis. Moreover, the FISH analysis indicated the abundant expression of cytoplasmic circ_0001236 in chondrocytes (Fig. 3D–F). Next, we examined the expression pattern of circ_0001236 in normal chondrocytes and OA cartilage tissues by FISH. We found a significantly higher expression level of circ_0001236 in normal chondrocytes than in OA cartilage (Fig. 3G). Collectively, these data suggest that abnormal circ_0001236 expression may be related to abnormal chondrogenesis and degeneration.

### Role of exosomal circ_0001236 in MSCs during chondrogenesis

**Fig. 4:**
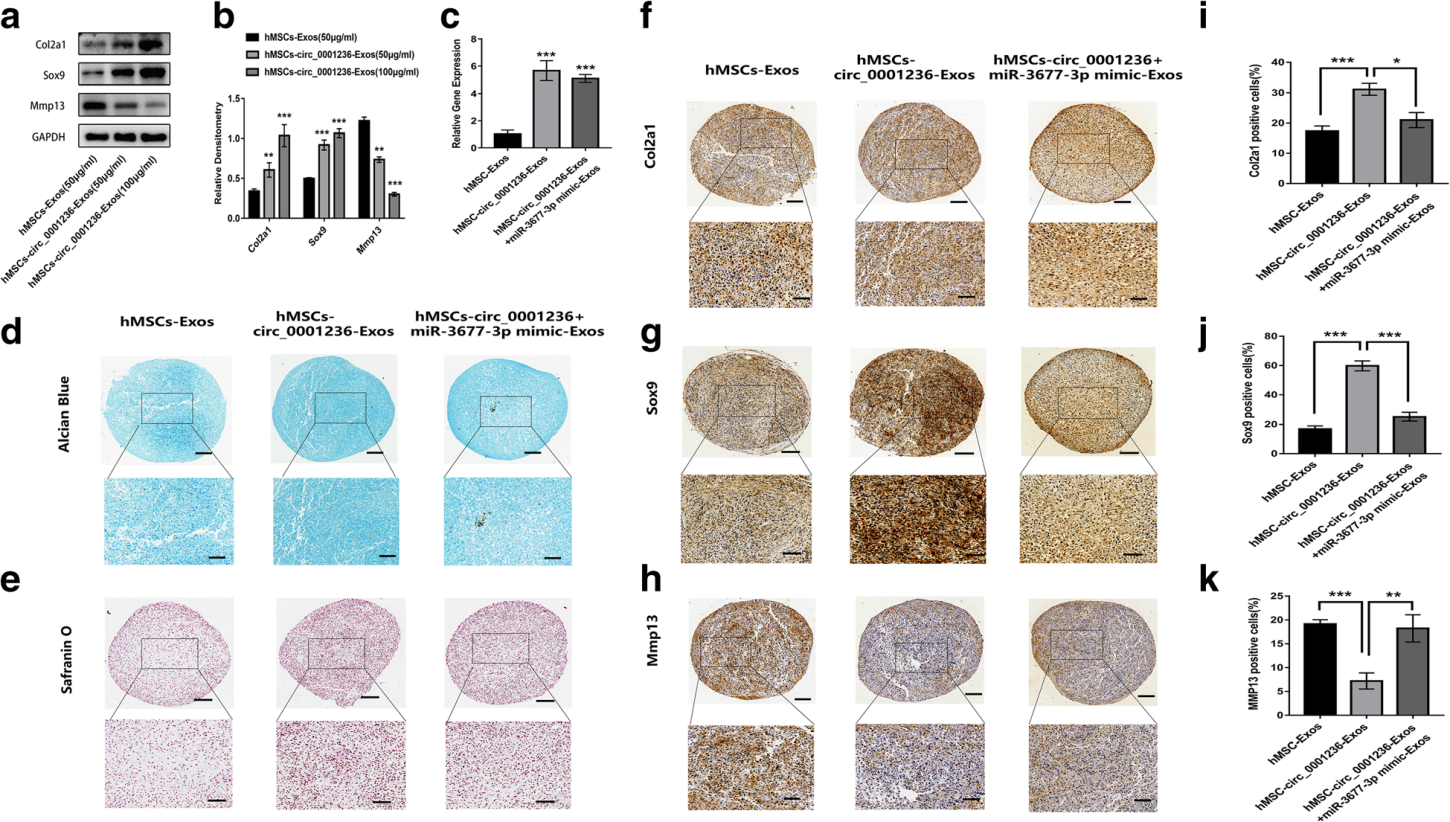
Exosomal circ_0001236 enhanced hMSC chondrogenesis. **a**, **b** hMSC micromass was treated with MSC-Exos and MSC-circ_0001236-Exos at different doses for 14 days. **c** The circ_0001236 level was detected in the MSC-Exos, MSC-circ_0001236-Exos, and MSC-circ_0001236+miR-3677-3p mimic-Exos groups. The MSC-circ_0001236-Exos (100 μg/mL) group showed stronger Alcian blue (**d**) and Safranin O (**e**) and higher Col2a1 (**f**, **I**) and Sox9 (**g**, **j**) expression, but lower MMP13 (**h**, **k**) expression level compared with those incubated with MSC-Exos and MSC-circ_0001236+miR-3677-3p mimic-Exos groups at days 21 with hMSCs induced to chondrogenesis. Moreover, the hMSC-circ_0001236-Exos inhibited MMP13 and enhanced the effect of Col2a1 and SOX9, which is affected by miR-3677-3p (**d**–**k**). Upper panels: × 100 magnification; lower panels: magnified view of the area enclosed by black boxes, × 400 magnification (scale bar, 50 μm). The experiment was repeated independently three times, and representative results are shown. GAPDH was used as endogenous control. *P < 0.05, **P < 0.01, ***P < 0.001

To further investigate the influence of circ_0001236 on chondrogenesis, the hMSC micromass was treated with 50 μg/mL MSC-Exos, 50 μg/mL MSC-circ_0001236-Exos, and 100 μg/mL MSC-circ_0001236-Exos for 14 days during chondrogenesis. We found that the 100 μg/mL MSC-circ_0001236-Exos group showed higher Sox9 and Col2a1 expression than MSCs incubated with 50 μg/mL or 100 μg/mL exosomes (Fig. 4A, B). Next, we co-cultured 100 μg/mL MSC-Exos, 100 μg/mL MSC-circ_0001236-Exos, and 100 μg/mL MSC-circ_0001236 + miR-3677-3p mimic-Exos with the MSC micromass for 21 days. Firstly, we detected the circ_0001236 level in different groups (Fig. 4C). Then, Alcian blue and Safranin O staining revealed that MSC-circ_0001236-Exos enhanced the chondrogenesis of MSCs, while MSC-circ_0001236 + miR-3677-3p mimic-Exos attenuated chondrogenesis (Fig. 4D, E). In order to comprehensively understand the effect of exosomal circ_0001236 on chondrogenesis, the chondrogenic markers Col2a1 (Fig. 4F, I), Sox9 (Fig. 4G, J), and cartilage degeneration marker MMP13 (Fig. 4H, K) were assessed in the three groups by IHC. Overexpression of exosomal circ_0001236 was found to increase Col2a1 and Sox9 expression, while miR-3677-3p mimic-Exos suppressed the expression of chondrogenic markers. These results suggested that exosomal circ_0001236 could promote the chondrogenic potential of MSCs; however, the effect is limited by miR-3677-3p. These data indicate that exosomal circ_0001236 may serve as a sponge for miR-3677-3p to promote Sox9 and Col2a1 expression and enhance chondrogenic differentiation.

### Exosomal circ_0001236 maintains the function of articular chondrocytes

**Fig. 5:**
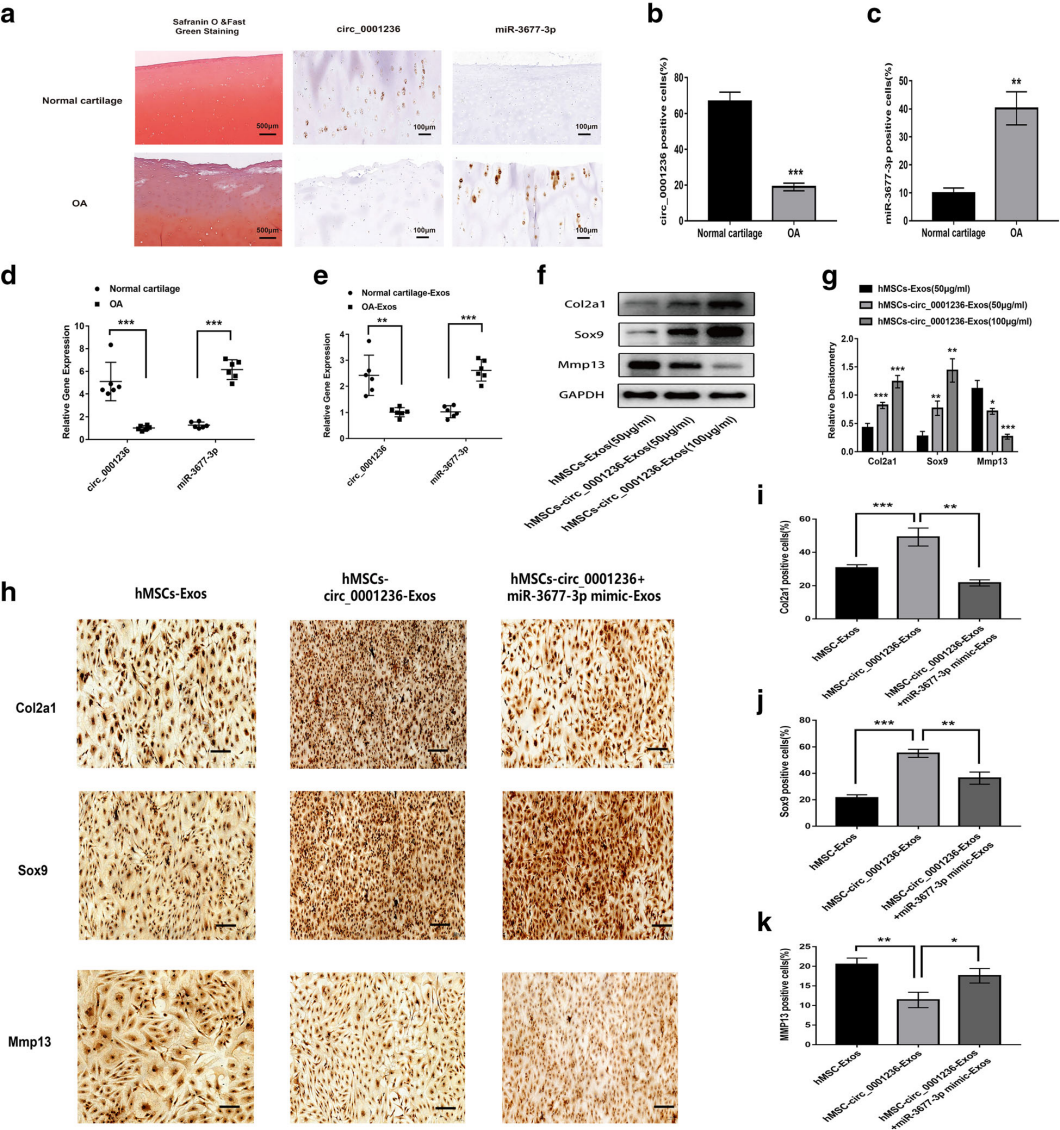
Exosomal circ_0001236 promotes cartilage-specific gene expression by sponging miR-3677-3p in human chondrocytes. **a**–**c** Expression of circ_0001236 and miR-3677-3p in healthy and OA cartilage by fluorescence in situ hybridization. **d**, **e** Relative exosomal circ_0001236 and miR-3677-3p expression levels in healthy and OA cartilage tissue and secreted exosomes were determined by qRT-PCR. GAPDH was used as endogenous control. Each dot represents a value from a single experiment involving a single donor. **f**, **g** The protein expression of Col2a1, Sox9, and MMP13 in OA chondrocytes were treated by MSC-circ_0001236-Exos (50 μg/mL, 100 μg/mL) and MSC-Exos (50 μg/mL). GAPDH was used as an endogenous control in western blotting. Immunohistochemistry analysis of Col2a1 (**h**, **i**), Sox9 (**h**, **j**), and MMP13 (**h**, **k**) is shown in OA chondrocytes, which were treated with hMSC-Exos, hMSC-circ_0001236-Exos, and hMSC-circ_0001236+miR-3677-3p mimic-Exos. The immunohistochemistry results were consistent with the western blot analysis. The experiment was repeated independently three times, and representative results are shown. GAPDH and U6 were used as endogenous control. *P < 0.05, **P < 0.01, ***P < 0.001

To determine the effect of exosomal circ_0001236 on chondrocyte function, we compared its expression levels in normal and OA cartilage-secreted exosomes and found significantly reduced expression in OA cartilage (Fig. 5A–E). However, OA cartilage exhibited higher levels of miR-3677-3p compared to that in normal cartilage (Fig. 5A–E). Then, we treated OA chondrocytes with 50 μg/mL MSC-Exos, 50 μg/mL MSC-circ_0001236-Exos, and 100 μg/mL MSC-circ_0001236-Exos for 48 h. We found that 100 μg/mL MSC-circ_0001236-Exos significantly upregulated the protein expression levels of Col2a1 and Sox9 but decreased MMP13 (Fig. 5F, G). Further, the 100 μg/mL MSC-Exos, 100 μg/mL MSC-circ_0001236-Exos, or 100 μg/mL MSC-circ_0001236 + miR-3677-3p mimic-Exos were co-cultured with OA chondrocytes for 72 h. The expression of Col2a1, Sox9, and MMP13 was assessed in the three groups by IHC to determine the effect of exosomal circ_0001236 on OA chondrocytes metabolism (Fig. 5H–K); overexpression of exosomal circ_0001236 was found to increase the expression of Col2a1 and Sox9, while the effect was reversed in the MSC-circ_0001236 + miR-3677-3p mimic-Exos group. These results suggest that exosomal circ_0001236 could promote cartilage-related specific matrix expression. However, the effect would be affected by miR-3677-3p. These data indicate that exosomal circ_0001236 may serve as a sponge for miR-3677-3p to promote Sox9 and Col2a1 expression and maintain cartilage function.

### Exosomal circ_0001236/miR-3677-3p/Sox9 axis regulates cartilage metabolic balance

**Fig. 6:**
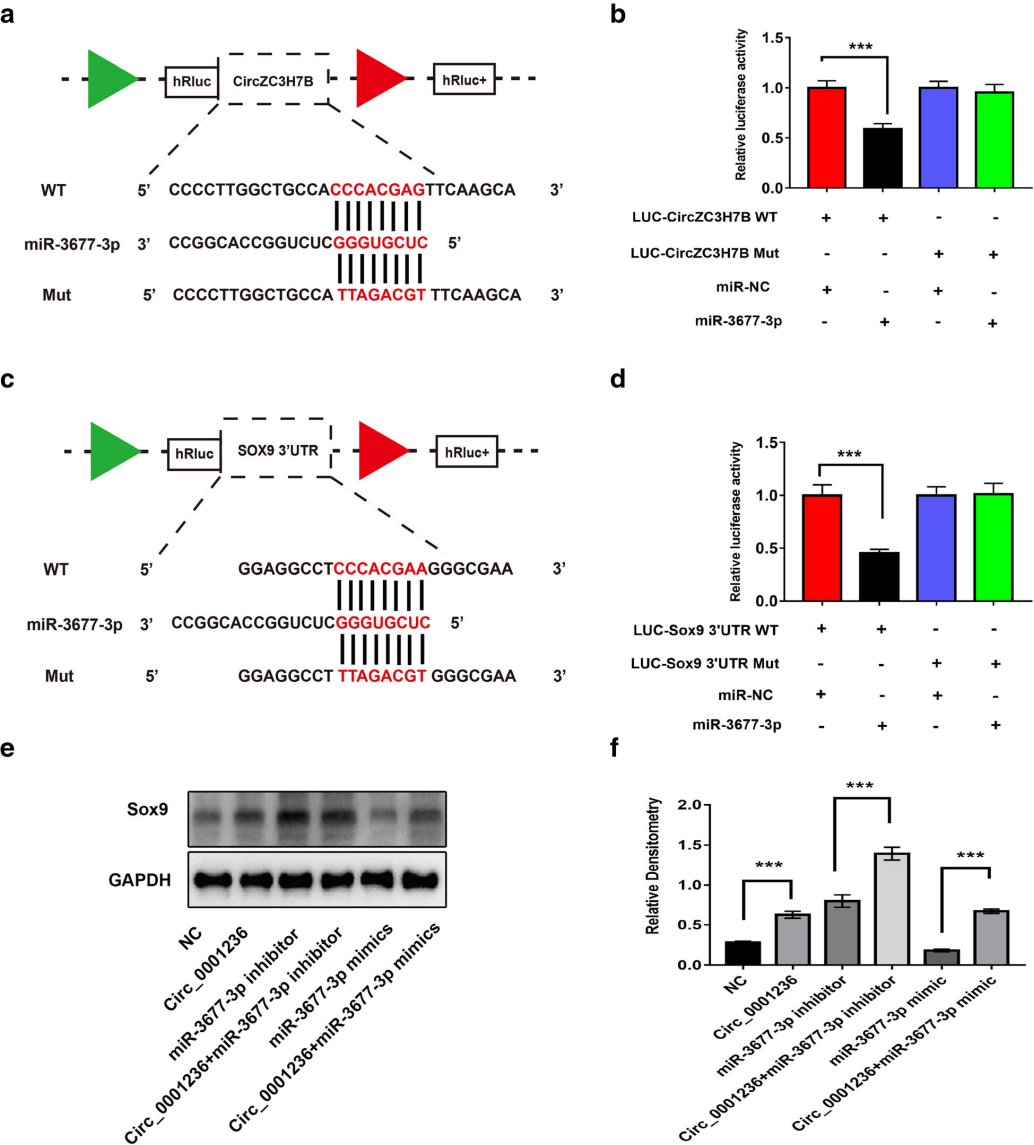
The exosomal circ_0001236/miR-3677-3p/Sox9 axis regulates cartilage metabolic balance. **a** Schematic illustration demonstrates the complementarity of the miR-3677-3p seed sequence with circ_0001236. **b** miR-3677-3p mimic or negative control (NC) was co-transfected with a luciferase reporter construct containing circ_0001236 wild-type (WT) or circ_0001236 mutant (MUT) into HEK293T cells. The luciferase reporter activities are shown. **c** Schematic illustration demonstrates the complementarity of the miR-3677-3p seed sequence with Sox9. **d** miR-3677-3p mimic or NC was co-transfected with a luciferase reporter construct containing Sox9 WT or Sox9 MUT into HEK293T cells, and the luciferase reporter activities of Sox9-MUT were markedly higher than that of Sox9 WT. Sox9 expression was regulated by hsa_circ_0001236 and miR-3677-3p (**e**, **f**). The experiment was repeated independently three times, and representative results are shown. GAPDH was used as endogenous control. ***P < 0.001

We performed qRT-PCR to determine the intracellular localization of circ_0001236 and founded that circ_0001236 was mainly localized in the cytoplasm (Fig. 3D–F). Previous studies showed that circRNAs in the cytoplasm might competitively bind to miRNAs and subsequently regulate their target genes by acting as miRNA sponges. Therefore, we speculated that circ_0001236 could target miRNAs to modulate their downstream functions. According to TargetScan (http://www.targetscan.org/vert_72/) and miRanda (http://sanderlab.org/tools/micrornas.html), we identified potential miRNAs targeted by circ_0001236; the results showed that miR-4636, miR-4487, miR-3677-3p, miR-762, and miR-4325 possess a binding site for circ_0001236. Next, we performed a luciferase screening assay to verify miRNA binding to circ_0001236. Interestingly, using co-transfection of miR-3677-3p mimic and the luciferase reporters containing circ_0001236 or circ_0001236 mutant vectors into HEK293T cells, we found that miR-3677-3p reduced the luciferase reporter activity by over 50% (Fig. 6A, B). Taken together, these results suggest that circ_0001236 might function as an miR-3677-3p sponge.

To further clarify the molecular mechanisms underlying the regulation of *Sox9* expression by miR-3677-3p, we analyzed the sequence of the 3′UTR of the human Sox9 mRNA. Bioinformatics software such as TargetScan and miRanda revealed that the 3′UTR of human Sox9 contains a potential miR-3677-3p-binding site (Fig. 6C). Luciferase reporter assays with the wild-type or mutant 3′UTR of Sox9 were performed in the presence or absence of miR-3677-3p. Transfection with miR-3677-3p resulted in reduced luciferase activity (indicating reduced transcription of Sox9) by miR-3677-3p binding to the wild-type 3′UTR, while the mutant 3′UTR sequence prevented this binding. This suggests that Sox9 is a target for miR-3677-3p-mediated repression (Fig. 6D). Sox9 expression was regulated by the circ_0001236 and miR-3677-3p (Fig. 6E, F).

### MSC-circ_0001236-Exos inhibits cartilage degradation in the DMM mouse model

**Fig. 7:**
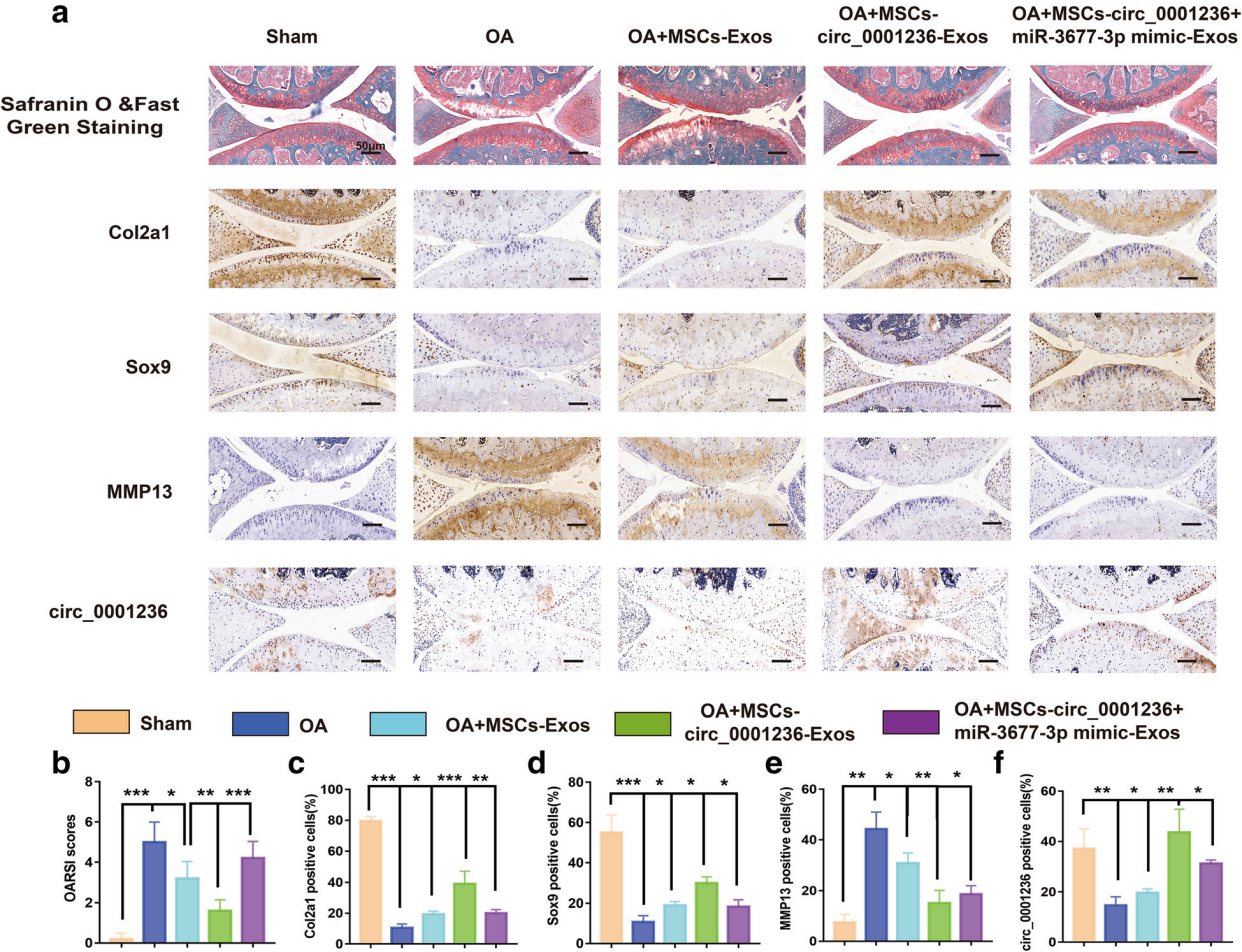
MSC-circ_0001236-Exos inhibits cartilage degradation in the DMM mouse model. **a** Safranin O and Fast Green staining, in situ hybridization of circ_0001236, and immunohistochemistry analysis of Col2a1, Sox9, and MMP13 are shown in the sections of the knee joints (*n* = 5 per group) from mice (scale bar, 50 μm). **b** OARSI scores of the knee joints of DMM mice of the five cohorts. Fold change of positive cell rates of Col2a1 (**c**), Sox9 (**d**), MMP13 (**e**), and circ_0001236 (**f**). *P < 0.05, **P < 0.01, ***P < 0.001

To gain further insight into the function of exosomal circ_0001236 in vivo, MSC-circ_0001236-Exos was administered to the DMM mice. The knee joints were harvested from five groups: control-, OA-, MSC-Exos-, MSC-circ_0001236-Exos-, and MSC-circ_0001236 + miR-3677-3p mimic-Exos-treated mice (Fig. 7A). They were subjected to Safranin O and Fast Green staining, which revealed that the MSC-Exos, MSC-circ_0001236-Exos, and MSC-circ_0001236 + miR-3677-3p mimic-Exos groups had a higher level of cartilage matrix than the OA group, and alos revealed that the Osteoarthritis Research Society International (OARSI) scores of the knee joints in the MSC-circ_0001236-Exos group were lower than those in the control group (Fig. 7B). Moreover, IHC showed that the expression levels of Col2a1 (Fig. 7C) and Sox9 (Fig. 7D) were increased, while that of MMP13 (Fig. 7E) decreased in the MSC-circ_0001236-Exos group compared with those in the OA and MSCs-Exos groups. We also showed the circ_0001236 level in these groups by hybridization in situ (Fig. 7A, F). Furthermore, MSC-circ_0001236 + miR-3677-3p mimic-Exos aggravated the degree of OA compared to the MSC-circ_0001236-Exos group. Taken together, these observations suggest that MSC-circ_0001236-Exos inhibited the progression of early OA and prevented severe damage to the knee articular cartilage in the DMM model by competitively adsorbing miRNA-3677-3p.

## Discussion

Exosomes are a critical bioactive component of MSC secretion that may be an alternative to MSC-based therapy [32, 33]. In the present study, we explored the ability of MSC-derived exosomal circRNAs to modulate OA pathogenesis and knee joint degeneration. Using circRNA microarrays, we identified several circRNAs that were differentially abundant in the exosomes isolated from MSCs that were induced to undergo chondrogenesis, suggesting that these factors may modulate cartilage differentiation and degeneration processes; upregulated circRNAs included circRNA_0025554, circRNA_0001236, circRNA_0035855, circRNA_0037422, circRNA_0091178, circRNA_0012265, circRNA_0039053, circRNA_0002643, circRNA_0082734, and circRNA_0076177. Notably, we identified a 5.29-fold upregulation of the exosomal circRNA_0001236 in chondro-differentiated MSC-Exos samples compared to its expression in the undifferentiated MSC-Exos; thus, we determined its association with OA disease activity. Exosomes derived from MSCs overexpressing circRNA_0001236 suppressed cartilage degeneration in chondrocytes and delayed OA progression in the DMM mouse model.

To evaluate the functional relevance of the circRNAs that were differentially expressed in chondro-differentiated MSC-Exos samples, we conducted GO and KEGG enrichment analyses on the target genes of these circRNAs. This approach revealed these exosomal circRNAs closely linked to key pathways associated with proliferation, migration, metabolism, and signal transduction. OA-related pathways identified via this approach include the PI3K-Akt, Wnt, mTOR, and AMPK signaling pathways. Therefore, we hypothesized that chondro-differentiated MSC-Exos might impact metabolic activity during chondrogenesis and cartilage degeneration. Our data supported this hypothesis, as do previous findings by Wu et al., who demonstrated that exosomes collected from infrapatellar fat pad MSCs suppressed the mTOR-autophagy pathway and promoted cartilage matrix expression in the DMM mouse model as compared with exosomes from non-stimulated cells [34]. Our results showed that exosomes derived from MSCs overexpressing circRNA_0001236 suppressed chondrocyte degeneration more effectively than exosomes derived from control MSCs, suggesting that these MSC-derived circRNA_0001236-overexpressing exosomes might be key regulators of chondrogenesis and chondrocyte degeneration.

Accumulating evidence has indicated the key mechanistic role of ceRNA networks in many diseases, including OA [35, 36]. Various RNAs, including circRNAs, long non-coding RNAs, and mRNAs, may function as ceRNAs in distinct physiological and pathophysiological conditions. For example, circSERPINE2 acts as an miR-1271-5p sponge, targeting miR-1271-5p and ERG in human chondrocytes in vivo and in vitro [36]. Similarly, circRNA.33186 contributes to the pathogenesis of osteoarthritis by sponging miR-127-5p [35]. Bioinformatics analysis of exosomal circRNA microarray data to explore the ceRNA regulation network revealed that ceRNA essentially operates in an miRNA-target mechanism [36]. The number of MREs is the key determinant of the range and strength of ceRNA regulation [37, 38]. Several validated ceRNA networks participate in the initiation and progression of various diseases [39, 40]. However, the overall pathophysiological contributions of exosomal circRNAs to OA remain largely unknown. In the present study, we hypothesized that circRNAs from MSC-derived exosomes might control cartilage differentiation and degeneration. We then demonstrated that exosomal circRNA_0001236 contains an miR-3677-3p target site that was validated by luciferase reporter assay. Furthermore, the expression of *Sox9*, an miR-3677-3p target, was positively regulated by exosomal circRNA_0001236. Therefore, we propose a mechanism wherein exosomal circRNA_0001236 acts as an miR-3677-3p sponge to promote ECM anabolism and suppress ECM catabolism, thereby delaying the progression of OA.

Furthermore, we also investigated the therapeutic utility of isolated MSC-circ_0001236-Exos in the treatment of DMM OA mice. We found that administration of circRNA_0001236-overexpressing exosomes into the articular cartilage was sufficient to suppress OA progression and enhance the regeneration of cartilaginous tissue, thereby preventing the development of severe damage in these DMM mice. However, this effect was reserved by miR-3677-3p. Therefore, we identified a new function of exosomal circRNA_0001236 in chondrogenesis and OA progression and thus demonstrated it to be a promising therapeutic target for OA treatment.

This study provides clear evidence that exosomal circRNA_0001236 can effectively suppress the degeneration of cartilaginous tissues. However, the shortcoming is that our sample size was small. Future studies, including those involving large cohorts with varying OA severity, are essential. We intend to further explore the signaling pathway activity in human chondrocytes to broaden our understanding of chondrogenesis regulation.

## Conclusions

We identified the exosomal circRNA_0001236–miR-3677-3p–Sox9 axis as a novel target to treat OA in the present study. circRNA_0001236-overexpressing exosomes may alleviate the catabolism of ECM, ultimately suppressing OA progression and enhancing the cartilage repair.

## Supplementary Information

**Additional file 1: **T**able S1.** circRNA expression profiles in exosomes before and after chondrogenic differentiation of MSCs. The expression profiles of circRNAs in exosomes before and after chondrogenic differentiation were detected using a circRNA microarray. SAM statistical software was used to identify differentially expressed circRNAs between undifferentiated MSC-Exos and chondro-differentiated MSC-Exos. Among the circRNAs that were consistently differentially expressed in all three paired samples, 12 were upregulated (fold change ≥ 2) in chondro-differentiated MSC-Exos compared to their expression levels in undifferentiated MSC-Exos.

**Additional file 2: Table S2.** Primers for quantitative real-time polymerase chain reaction (qRT-PCR).

## Data Availability

All data generated or analyzed during this study are included in this published article and additional files.

## Abbreviations

hMSC: Human bone marrow-derived MSC
DMEM: Dulbecco’s modified Eagle medium
MSC-Exos: Exosomes secreted by mesenchymal stem cells
MSC-circRNA_0001236-Exos: Exosomes secreted by circRNA_0001236-overexpressing mesenchymal stem cells
Sox9: SRY (sex-determining region Y)-box 9
