# Supplementary material for: Exosome-transported circRNA_0001236 enhances chondrogenesis and suppress cartilage degradation via the miR-3677-3p/Sox9 axis
Source: Stem Cell Res Ther. 2021 Jul 13;12:389. doi: 10.1186/s13287-021-02431-5 (PMC8278601; doi:10.1186/s13287-021-02431-5)
Supplement: Supplementary file 2 — Additional file 2: Table S2. Primers for quantitative real-time polymerase chain reaction (qRT-PCR). [file 13287_2021_2431_MOESM2_ESM.docx]

**Supplementary material table 2. Primers for quantitative real-time polymerase chain reaction (qRT-PCR)**

Gene primer sequence(5'-3')

hsa-circ_0001236-F ACTACCTGTGCAAAGCCAGA

hsa-circ_0001236-R CCCTCACGGTAGGTGTAGTC

hsa_circ_0025554-F CTAATCCACGCTGACCTCAA

hsa_circ_0025554-R ACATGTGAGGCCATAGCTGT

hsa_circ_0035855-F GCTGGAGATCGATTGCTTAC

hsa_circ_0035855-R CTACAAGTCTGCTAACTTCA

hsa_circ_0037422-F ACTACTGCAATGGCCATGCTTGA

hsa_circ_0037422-R gctgacacttgtcacaggttag

hsa_circ_0091178-F TTCTCCGTCAATGTTTCCAGCC

hsa_circ_0091178-R cgagccagttgatgctctgcac

hsa-miR-3677-3p-F CTCGTGGGCTCTGGCCACGGCC

hsa-GAPDH-F GCACCGTCAAGGCTGAGAAC

hsa-GAPDH-R TGGTGAAGACGCCAGTGGA

hsa-U6-F CTCGCTTCGGCAGCACA

hsa-U6-R AACGCTTCACGAATTTGCGT
